# P-856. Penicillin Allergy Prevalence and Association with Prescription of clostridioides difficile Infection-Associated Antibiotics within People Incarcerated in Wisconsin Department of Corrections

**DOI:** 10.1093/ofid/ofaf695.1064

**Published:** 2026-01-11

**Authors:** Rachel A Tam, Samuel Wilk, Kap Sum Foong, Lindsay Taylor, Alysse Wurcel

**Affiliations:** Boston Medical Center, Boston, Massachusetts; Tufts Medical School, Boston, Massachusetts; Tuft Medical Center, Tufts University School of Medicine, Boston, MA; Wisconsin Department of Public Health, Madison, Wisconsin; Boston Medical Center, Boston, Massachusetts

## Abstract

**Background:**

Unconfirmed penicillin allergy labels (PALs) have a negative impact on personal and public health due to the prescription of overly broad antibiotics which results in antimicrobial-resistant infections. As most people with PALs are not truly allergic, there has been increased implementation of penicillin allergy delabeling initiatives. Although penicillin allergy delabeling is not yet routine in carceral settings, Wisconsin (WI) Department of Corrections (DOC) is striving to increase access to it for the state’s incarcerated population.

**Table 1:**
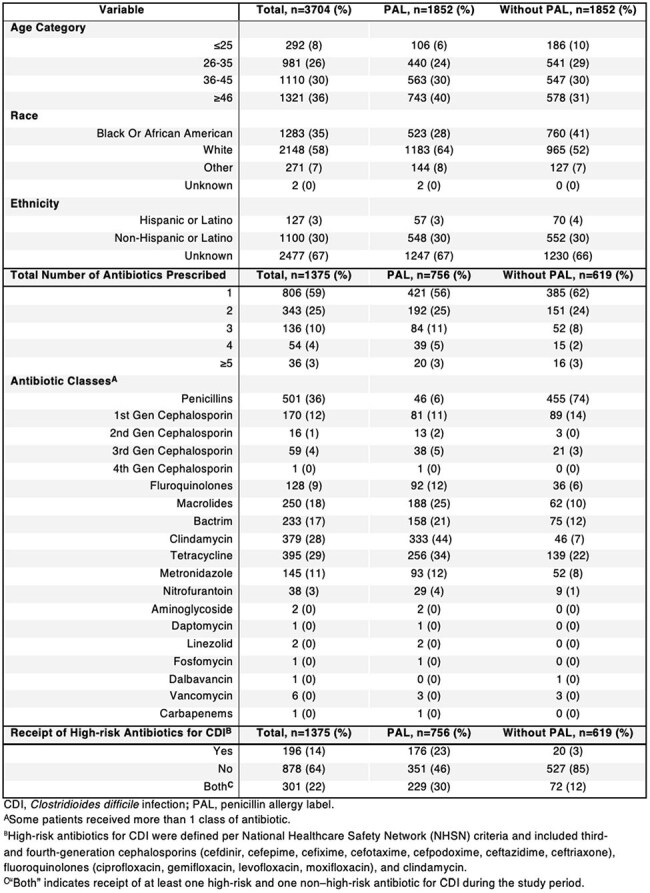
Demographic Characteristics, Antibiotic Use, and Receipt of High-Risk Antibiotics for Clostridioides difficile Infection, Stratified by Penicillin Allergy Label Status

**Figure:**
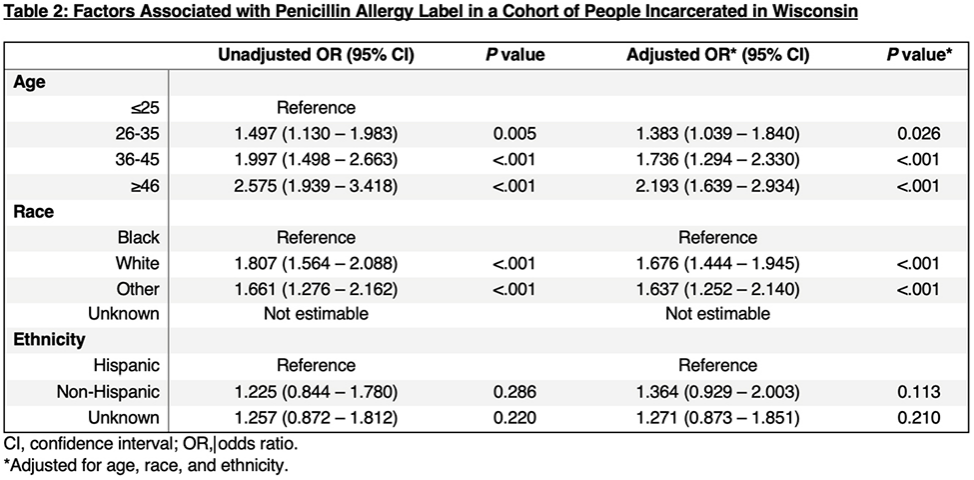

**Methods:**

The prevalence of PALs in individuals incarcerated within the state was calculated from the 2021-2023 WI DOC Census. We created a retrospective cohort of people incarcerated in WI DOC with all individuals with PALs and an equal number of randomly selected individuals without PALs. We collected age, race, ethnicity, PALs, and all prescribed antibiotics – categorized as high-risk or non-high-risk for *Clostridioides difficile* infection (CDI). Conditional logistic regression analysis was performed to assess (1) the association between demographic characteristics and PAL and (2) predictors of any antibiotic prescription. Multivariable regression identified predictors of high-risk antibiotic prescriptions for CDI.

**Figure:**
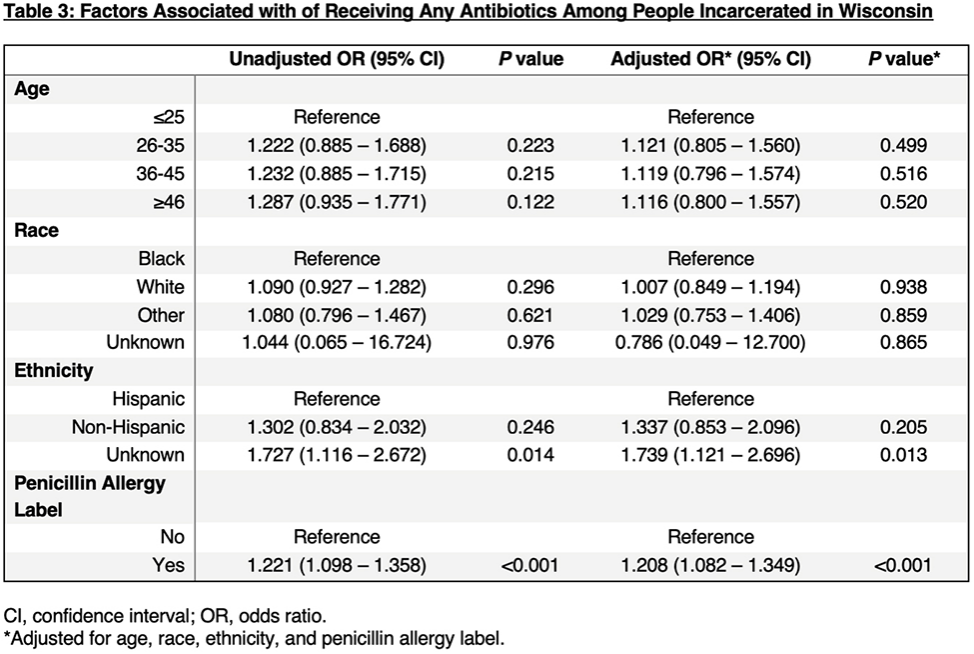

**Figure:**
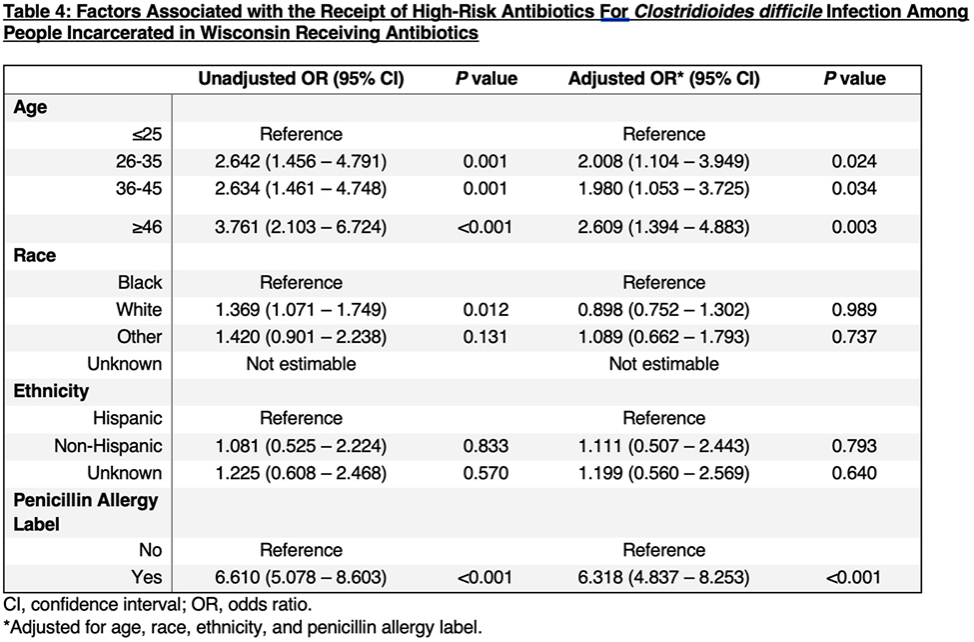

**Results:**

Of the total WI DOC incarcerated population (n=20,483),1,852 people (9%) had PALs. People with PALs were more likely to be 46 years or older (adjusted odds ratio [aOR], 2.193; 95% confidence interval [CI], 1.639–2.934), White (aOR, 1.676; 95%CI, 1.444–1.945), and non-Hispanic (aOR, 1.364, 95% CI, 0.929-2.003) (Table 2). People with PALs were more likely to be prescribed an antibiotic (aOR, 1.208; 95% CI, 1.082-1.349; Table 3) and a high-risk antibiotic for CDI (aOR, 6.318; 95% CI, 4.837-8.253; Table 4).

**Conclusion:**

Our study is the first to examine the prevalence of PALs in a carceral setting. These findings demonstrate that people with PALs were more likely to be White/non-Hispanic, and 46 years or older. Those with PALs were more likely to be prescribed any antibiotic and a high-risk antibiotic for CDI. Collaboration with stakeholders in correctional settings is essential to support effective antibiotic stewardship implementation.

**Disclosures:**

All Authors: No reported disclosures

